# A Predictive Model Facilitates Early Recognition of Spinal Epidural Abscess in Adults

**DOI:** 10.5811/westjem.2017.35778

**Published:** 2018-02-12

**Authors:** Andrew W. Artenstein, Jennifer Friderici, Paul Visintainer

**Affiliations:** * Baystate Health, Department of Medicine, Springfield, Massachusetts; † University of Massachusetts Medical School-Baystate, Springfield, Massachusetts

## Abstract

**Introduction:**

Spinal epidural abscess (SEA), a highly morbid and potentially lethal deep tissue infection of the central nervous system has more than tripled in incidence over the past decade. Early recognition at the point of initial clinical presentation may prevent irreversible neurologic injury or other serious, adverse outcomes. To facilitate early recognition of SEA, we developed a predictive scoring model.

**Methods:**

Using data from a 10-year, retrospective, case-control study of adults presenting for care at a tertiary-care, regional, academic medical center, we used the Integrated Discrimination Improvement Index (IDI) to identify candidate discriminators and created a multivariable logistic regression model, refined based on p-value significance. We selected a cutpoint that optimized sensitivity and specificity.

**Results:**

The final multivariable logistic regression model based on five characteristics –patient age, fever and/or rigor, antimicrobial use within 30 days, back/neck pain, and injection drug use – shows excellent discrimination (AUC 0.88 [95% confidence interval {0.84, 0.92}]). We used the model’s β coefficients to develop a scoring system in which a cutpoint of six correctly identifies cases 89% of the time. Bootstrapped validation measures suggest this model will perform well across samples drawn from this population.

**Conclusion:**

Our predictive scoring model appears to reliably discriminate patients who require emergent spinal imaging upon clinical presentation to rule out SEA and should be used in conjunction with clinical judgment.

## INTRODUCTION

The incidence of spinal epidural abscess (SEA), a highly morbid and potentially lethal deep tissue infection of the central nervous system, has risen significantly over the past decade.^1^, ^2^ Our tertiary care institution has experienced an increase of more than 200%, from 2.5 to 8 cases per 10,000 hospital admissions since 2005.^3^ Although the reasons are not clearly defined, various factors, such as an expanded, comorbidly ill, aging population, and procedures or behaviors predisposing to bacteremia, have been posited to contribute to the increased incidence of SEA.

We recently reported the results of a large, case-control study of SEA over the previous decade in a single, tertiary-care, regional, academic medical center to assess possible changes in the epidemiology, risk factors, and clinical manifestations of this infection in order to identify features that could potentially facilitate its early clinical recognition.^3^ Because SEA may rapidly and unpredictably evolve to irreversible neurologic injury and diagnostic delays remain common,^4^ our goal was to use these data to inform a discrimination model that could be employed at the time of initial clinical presentation to prioritize potential cases for expeditious, advanced imaging to optimize patient outcomes.

## METHODS

### Study Design

The design and selection criteria for cases and controls have been previously described.^3^ To ensure clinical relevance, the case and control groups were drawn from patients who presented with findings that either raised concern for SEA or who underwent a “rule-out” evaluation; magnetic resonance imaging (MRI) or computed tomography and microbiologic data were used to assign patients to the appropriate group. Baystate Medical Center (BMC), a 720-bed tertiary-care, regional, academic medical center currently serving a population of approximately 850,000 people in western Massachusetts experiences more than 33,000 annual adult discharges with a corresponding case-mix index of 1.72, indicating high severity and complexity of its inpatients relative to their diagnosis related group. Encounters were coded as “confirmed” SEA if there was a radiologist-confirmed finding of an epidural lesion on advanced imaging with a positive culture from lesion or blood; “probable” if there was a radiologist-confirmed epidural lesion in the absence of positive cultures from lesion or blood; and “control” if no lesion was identified by the radiologist on the imaging study. This study was approved by the institutional review board.

### Statistical Analysis

We preliminarily evaluated baseline comparison between cases and controls using univariable analyses (t-tests, Fisher’s exact test) and direct visualization methods (coefficient plots, LOWESS curves). Because our goal was to develop a discrimination model, we used the Integrated Discrimination Improvement Index (IDI) to identify candidate discriminators.^5^ The IDI represents the degree to which a candidate variable increases the event probability in cases, while decreasing the event probability in controls, thus discriminating cases from non-cases.

We selected candidate predictive factors if they were immediately discernible upon clinical presentation and if their univariable IDI was >0.02, suggesting meaningful discrimination properties. To reduce bias in the prediction model, candidate variables had to have at least 20 events to be considered.^6^ The multivariable logistic regression model initially included all candidate variables and was then refined using a backwards selection process, with a p-value for removal of 0.05. We used Youden’s J^7^ to identify the cutpoint that maximized sensitivity and specificity. Areas under the receiver operator curve (AUC) of the full- vs. restricted-models were compared using previously described methods.^8^ We assessed model fit using the Hosmer-Lemeshow goodness of fit^9^ and Stukel^10^ tests. Because measures of model validation may be overly optimistic when derived on the sample used for coefficient estimation, we generated bootstrapped validation measures.^11^,^12^ We used Stata 14.2 (StataCorp LLC, College Station TX) and R (http://www.R-project.org/) for analyses.

**Population Health Research Capsule**

What do we already know about this issue?

Spinal epidural abscess (SEA) is a highly morbid and potentially lethal infection of rising incidence that is often associated with diagnostic delays. Early diagnosis appears to improve outcomes.

What was the research question?

Can a predictive scoring model be developed that may facilitate the early diagnostic recognition of SEA?

What was the major finding of the study?

Through a large, controlled data set, five factors apparent upon clinical presentation showed robust discrimination of cases.

How does this improve population health?

The predictive model appears to reliably discriminate patients who require emergent spinal imaging upon ED presentation to rule out SEA and should be used in conjunction with clinical judgment.

## RESULTS

We identified 162 cases (“confirmed” and “probable”), representing 64.8% of 250 admissions in which SEA was deemed to be a significant diagnostic consideration upon clinical presentation, and a “rule-out” process ensued. Demographic and clinical characteristics of the sample overall (i*.*e*.*, cases and controls) and by case status revealed several variables of potential significance (p-values < 0.05) with their corresponding IDI values (Table 1). Interestingly, several factors that have been previously reported to confer risk for SEA in uncontrolled studies,^1^,^2^, such as diabetes mellitus or the presence of focal neurologic deficits, were not found to differentiate cases from controls.

Nine characteristics met initial criteria as candidate variables based on statistical significance: age (with the quadratic representation); fever/rigor (fever defined as self-reported or measured temperature of ≥100.4°F); non-traumatic back or neck pain; receipt of antimicrobials within 30 days of admission; a previous ED visit within 30 days of admission,; injection drug use; morbid obesity (defined as BMI ≥35 mg/kg^2^); radicular pain; and alcohol abuse. Four of these characteristics (radicular pain, previous healthcare visit within 30 days, obesity, and alcohol abuse) were subsequently removed for poor multivariable discrimination based on IDI (Table 1).

### Multivariable Model

The final prediction model comprising five factors is shown in Table 2. Model fit was improved significantly with the addition of a quadratic term for age, as risk was reduced in the youngest and oldest patients. The multivariable model achieves an AUC of 0.88 (95% CI [0.84, 0.92]) (Figure a). There was no evidence of significant departure from fit (H-L test, p=0.39; Stukel test = 0.37). Fever/rigor was the strongest clinical predictor of case status in the multivariable model, contributing eight percentage points to its AUC.

To aid clinical decision-making we developed a scoring tool (Table 2) by modifying the final regression model in two ways: age and its quadratic term were represented using seven categorical variables reflecting 10-year age intervals; and the final logistic model beta coefficients were rounded to the nearest integer to facilitate scoring. The quadratic term for age indicates that the risk of SEA is not constant as age increases; risk of SEA generally increases with age and then attenuates somewhat in the oldest age group. By representing age in categories, we can incorporate this non-linearity into the risk score. Applying the tool to the sample total scores ranged from 0–11. The mean score for cases was 7.6 (± 1.7) with a range of 3–11. The mean score for controls was 4.9 (± 1.7) with a range of 0–9. The difference in the means was significant (p < 0.001). The tool showed similar discriminating properties to the final regression model. The AUC for the tool was 0.88 (95% CI [0.84, 0.92]). Setting a cutpoint of six on our scoring tool resulted in a sensitivity of 89% (95% CI [83%, 93%]) and a specificity of 63% (95% CI [52%, 73%]). At this cutpoint, with a sample prevalence of 65%, the positive predictive value (PPV) was 81% (95% CI [75%, 87%]), and the negative predictive value (NPV) was 75% (95% CI [64%, 85%]). Finally, to increase sensitivity we considered a cutpoint of five or greater as positive: sensitivity was 96% (95% CI [92%, 99%]) and specificity was 34% (95% CI [24%, 45%]). The PPV was 73% (95% CI [665. 79%]) and the NPV was 83% (95% CI [63%, 94%]).

We explored misclassification of the method by examining extreme scores of cases and non-cases. Among cases that presented with the lowest scores were three patients above the age of 60 who presented with none of the clinical features (injection drug use, fever/rigor, back/neck pain, anti-microbial use with 30 days). Among non-cases that scored highest were patients who presented with two or three of the clinical features and were above the age of 30. There were no non-cases who presented with all four of the clinical features. Five cases (3.1%) presented with all four of the clinical features.

### Model Validation

The model demonstrated excellent discrimination between cases and non-cases (Figure a). To account for biased estimates of model fit and validation, bootstrapped estimates were derived on 1,000 model runs; bias-adjusted estimates of model validity suggested good model fit (Figure b). The model shows excellent agreement between predicted and observed probabilities, particularly at the higher probability levels but slightly underestimates the observed probability at the lowest probability levels. The corresponding c-statistic was 0.86, suggesting excellent discrimination. The model has good predictive strength as indicated by Nagelkerke’s *R^2^* of 0.47.^11^

## DISCUSSION

The incidence of SEA has progressively increased over the past several decades;^1^,^2^ it has more than tripled over the past decade at our institution and with this, the risk of serious neurologic morbidity has risen commensurately.^3^ Although early recognition is essential to preventing irreversible neurologic deficits, diagnostic delays are common.^4^ Perhaps the best opportunity for early diagnosis occurs at the time of initial clinical presentation. The majority of patients with SEA present to a healthcare facility with clinical manifestations more than once within 30 days of diagnosis; in most cases, these visits are to the ED.^3^,^4^ Therefore, a clinical scoring tool that could reliably discriminate potential cases and stratify them for emergent spinal imaging at the time of initial clinical presentation would be of value to optimize clinical outcomes in SEA, while also providing stewardship and prioritization of imaging resources.

Diabetes mellitus and other, chronic, co-morbid illnesses are often listed as predisposing risks for SEA in the extant literature.^1^,^2^ Recently reported data from our institution, representing the largest, single-site, published SEA case series (162 cases) with an attendant control group, failed to confirm these as risk factors.^3^

Our predictive model shows excellent agreement between observed and predicted probabilities (Figure). Although further refinement of the model may improve these classification characteristics, maximizing sensitivity and specificity may not be optimal for clinical application. Because of the potentially severe consequences of delaying the diagnosis of SEA, it may be more clinically exigent to maximize sensitivity and thereby sacrifice some degree of specificity. Using our scoring tool with a cutpoint at six, sensitivity appeared to be optimized (89%) at the expense of a modest decrease in specificity (63%). At a higher cutpoint of seven, sensitivity – the ability of the model to correctly detect positives – was reduced to 77% (95% CI [69%, 83%]). At this cutpoint the PPV was 93% (95% CI [87%, 96%]), but the NPV was only 67% (95% CI [58%, 76%]). At a lower cutpoint of five, the modest increase in sensitivity was associated with substantial decrement in specificity (34%) that was felt to be too low for clinical utility.

A previous study evaluated a clinical decision guideline based on elevated serum inflammatory markers to determine the need for advanced spinal imaging in patients potentially at risk for SEA in the ED^13^ Although use of the guideline at one institution appeared to reduce diagnostic delays as compared with historical controls, detailed information on the use of MRI was not provided. Additionally, the guideline relies on laboratory testing, which could introduce further delays. We sought to develop a clinically relevant model that was based exclusively on epidemiologic and clinical features that are apparent on initial clinical presentation in order to appropriately triage MR spinal imaging and to reliably facilitate the early recognition of SEA as distinct from other potential spinal pathologies.

## LIMITATIONS

Our model has several limitations. The data were drawn from a retrospective, 10-year cohort; thus, the model is based on clinical variables collected at the time of admission or shortly thereafter and available in the record. Although we strove to collect complete data on all variables, it is possible that some potentially useful factors were not considered. Additionally, to optimize the clinical utility of our model, we purposefully limited it to clinical data that would be apparent on initial presentation. We did not consider serum inflammatory markers or other laboratory data in this category. Review of erythrocyte sedimentation rate (ESR) levels in our cohort revealed that this marker was only obtained in approximately 60% of the patients, and the vast majority of these were cases, suggesting that ESR was requested only in the clinical presentations that were highly suspicious for SEA.

Another important limitation of our model is that it was derived from data from a decade-long, retrospective cohort of patients with a confirmed SEA case prevalence of 65% from a single institution; thus, the clinical presentations in this cohort raised at least some level of suspicion for the diagnosis. This scoring model may therefore only be relevant when SEA is reasonably suspected. Our work underscores the known importance of clinical judgment in suspecting the diagnosis of SEA;^1^,^3^ the objective model detailed herein serves to complement this subjective consideration.

Because our institution is a regional, tertiary-care, academic medical center, it is also important to determine whether our data can be extrapolated to other care settings. A prospective evaluation and validation of this model is needed to understand whether it may be useful in an unselected sample of patients presenting for medical attention with a constellation of symptoms and/or signs potentially warranting investigation for SEA. Such an evaluation may also determine if the model can be substantially improved by incorporating additional data that could be ascertained within a short time frame after ED presentation.

## CONCLUSION

We have developed a scoring model that appears to reliably discriminate patients who require emergent spinal imaging upon clinical presentation to rule out SEA. It is hoped that our work contributes to raising awareness of this increasingly important, highly morbid, central nervous system infection, and that further enhancement of our model through future prospective validation, prior to its adoption into clinical practice, may limit adverse outcomes from this illness.

## Figures and Tables

**Figure:**
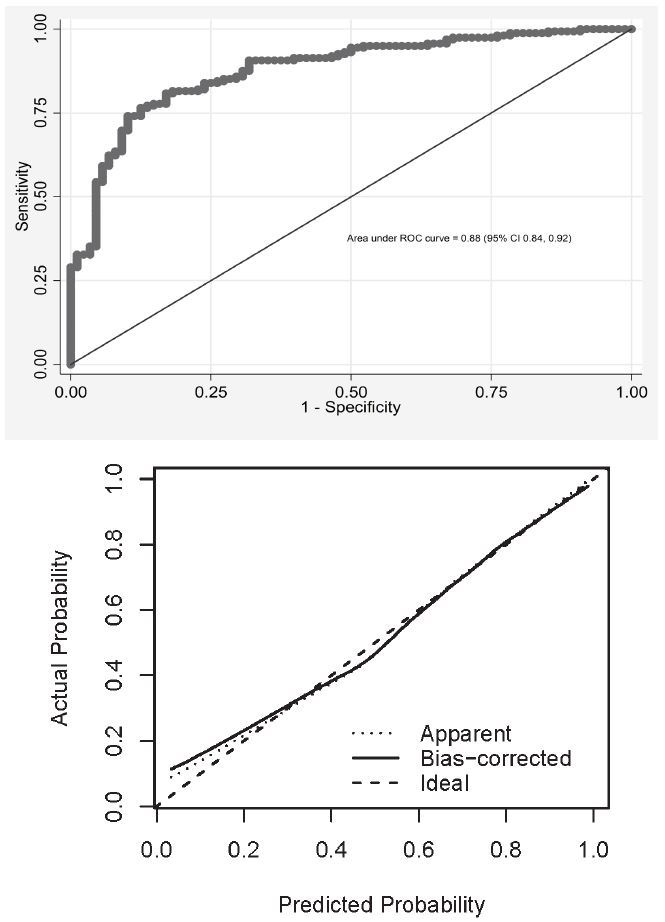
a) ROC plot of final prediction model (Top) b) calibration plot of final model (bottom).

**Table 1:** Characteristics of study sample: overall and by study group.

Factor	Overall (n=250)	Case (n=162)	Control (n=88)	P value*	IDI
Age in Years, mean±SD	59.7±15.6	58.8±13.8	61.4±18.3	< 0.001#	0.23
Age^2^, mean±SD	3810.7±1835	3652.8±1634	4101.5±2137		
Fever and/or Rigor	113 (45.2%)	101 (62.3%)	12 (13.6%)	<0.001	0.22
Back and/or Neck Pain	203 (81.2%)	149 (92.0%)	54 (61.4%)	<0.001	0.14
Antimicrobials w/in 30d	63 (25.2%)	57 (35.2%)	6 (6.8%)	<0.001	0.10
Injection Drug Use	37 (14.8%)	33 (20.4%)	4 (4.5%)	<0.001	0.05
Previous ED Visit	108 (43.2%)	82 (50.6%)	26 (29.5%)	0.001	0.04
BMI>35 mg/kg2	43 (17.2%)	34 (21.0%)	9 (10.2%)	0.04	0.02
Radicular Pain	55 (22.0%)	42 (25.9%)	13 (14.8%)	0.05	0.02
Alcohol Abuse	38 (15.2%)	31 (19.1%)	7 (8.0%)	0.03	0.02
Paresthesia	94 (37.6%)	55 (34.0%)	39 (44.3%)	0.13	0.01
Diabetes Mellitus	76 (30.4%)	55 (34.0%)	21 (23.9%)	0.11	0.01
End Stage Renal Disease	15 (6.0%)	13 (8.0%)	2 (2.3%)	0.09	0.01
Spinal Injection w/in 30d	10 (4.0%)	9 (5.6%)	1 (1.1%)	0.10	0.01
HIV/AIDS	4 (1.6%)	1 (0.6%)	3 (3.4%)	0.13	0.01
Focal Weakness	113 (45.2%)	68 (42.0%)	45 (51.1%)	0.18	< 0.01
Altered Mental Status	58 (23.2%)	41 (25.3%)	17 (19.3%)	0.35	< 0.01
Urinary Retention	36 (14.4%)	20 (12.3%)	16 (18.2%)	0.26	< 0.01
Immune Comp. (Non-HIV)	17 (6.8%)	12 (7.5%)	5 (5.7%)	0.79	< 0.01
Bowel/Bladder Incontinence	15 (6.0%)	10 (6.2%)	5 (5.7%)	1.00	< 0.01

* Fisher’s exact or independent samples t-test;

# Likelihood ratio test

*IDI*, Integrated Discrimination Improvement Index; *BMI*, body-mass index; *SD*, standard deviation

**Table 2:** Final prediction model for spinal epidural abscess.

Characteristics	Prediction model				Clinical score

	Log-odds	Odds ratio	95% CI	p-value	
Fever and/or rigor	2.31	10.09	4.53, 22.44	< 0.001	2
Antimicrobial use within 30d	1.98	7.25	2.55, 20.61	< 0.001	2
Back and/or neck pain	1.80	6.04	2.13, 17.11	0.001	2
Injection drug use	1.61	5.00	1.70, 14.67	0.003	2
Age	0.23	1.26	1.08, 1.48	0.004	-----
Age^2^	-0.002	0.998	0.996, 0.999	0.008	-----
20 to < 30					0
30 to < 40					3
40 to < 50					3
50 to < 60					4
60 to < 70					3
70 to < 80					4
80+					3
